# A Randomized Controlled Trial of a Digital Patient Decision Tool to Increase Sexually Transmitted Infection Testing in the Emergency Department

**DOI:** 10.1016/j.annemergmed.2026.03.016

**Published:** 2026-05-15

**Authors:** Lauren S. Chernick, Talia Adler, Jaciara De Souza, Christina N. Franqui, Lisa-Pierre Tchoungui, Simian Huang, Marc A. Probst, Delivette Castor, Jason Zucker

**Affiliations:** Department of Emergency Medicine, Columbia University Medical Center, New York, NY; Department of Population and Family Health, Mailman School of Public Health, Columbia University Medical Center, New York, NY; Department of Emergency Medicine, Columbia University Medical Center, New York, NY; Department of Emergency Medicine, Columbia University Medical Center, New York, NY; Department of Emergency Medicine, Columbia University Medical Center, New York, NY; Department of Emergency Medicine, Columbia University Medical Center, New York, NY; Division of Infectious Diseases, Department of Medicine, Columbia University Irving Medical Center, New York, NY; Department of Emergency Medicine, Columbia University Medical Center, New York, NY; Division of Infectious Diseases, Department of Medicine, Columbia University Irving Medical Center, New York, NY; Department of Epidemiology, Columbia University Mailman School of Public Health, New York, NY; Division of Infectious Diseases, Department of Medicine, Columbia University Irving Medical Center, New York, NY; Department of Pediatrics, Columbia University Medical Center, New York, NY.

**Keywords:** Sexually transmitted infection testing, Emergency department, Mobile health intervention, Shared decisionmaking, Adolescent sexual health

## Abstract

**Study objective:**

To evaluate the efficacy of a digital patient decision aid, Sexually Transmitted Infection Check in the Emergency Room (STIckER), in increasing sexually transmitted infection testing among adolescents and young adults in the emergency department (ED).

**Methods:**

We conducted a randomized controlled trial in adult and pediatric EDs at a large urban academic medical center. We randomized health care clinicians to deliver either the STIckER digital decision aid or usual care to sexually active adolescents and young adults patients aged 14 to 24 years. The tool, developed through human-centered design, provided personalized sexually transmitted infection testing recommendations and values clarification exercises via smartphone. The primary outcome was documentation of gonorrhea/chlamydia testing. Secondary outcomes included extragenital testing, patient-reported decisionmaking measures, and implementation. We conducted descriptive statistics of patient-level and health care provider–level outcomes by study arms. We estimated the efficacy of STIckER using log-binomial regression.

**Results:**

We randomized 44 health care clinicians and enrolled 139 adolescents and young adult participants (median age 19 years). Participants in the STIckER arm were 1.76 times more likely to receive gonorrhea/chlamydia testing (95% confidence interval, 1.10 to 3.00) and 4.56 times more likely to receive pharyngeal testing (95% confidence interval, 1.30 to 28.66). Participants in the STIckER arm reported greater clarity about testing options and higher satisfaction with ED care. Adolescents and young adults and health care clinicians rated the tool as highly acceptable, appropriate, and feasible.

**Conclusion:**

A digital, patient-centered decision aid increased sexually transmitted infection testing among adolescents and young adults in the ED. STIckER is feasible for integration into ED clinical workflows and may represent a scalable strategy to expand equitable sexual health screening in acute care settings.

## INTRODUCTION

### Background

Sexually transmitted infections, including extragenital (oropharyngeal and anorectal) infections, remain a major public health challenge in the United States.^1^ Adolescents and young adults aged 15 to 24 years account for nearly half of all chlamydia and gonorrhea cases.^1^ Over the past decade, there has been a 33% increase in overall sexually transmitted infection cases, with more than 2.4 million infections reported nationwide.^1^ Sexually transmitted infection burden may be underestimated because comprehensive screening, including extragenital testing, is infrequently performed.^2,3^

Emergency departments (EDs), which serve more than 19 million adolescents and young adults annually, are a critical access point for care, particularly for those from socioeconomically disadvantaged communities.^4,5^ Data show that many adolescents and young adults who use the ED for care often do not seek care elsewhere and, if they do, rarely have confidential conversations with their health care clinicians about their sexual health.^5,6^ However, multiple barriers contribute to infrequent screening in the ED, particularly for extragenital sites, such as time constraints, limited clinician training, competing clinical priorities, and lack of awareness of sexually transmitted infection screening guidelines.^7^ Addressing this gap presents an important opportunity to enhance sexually transmitted infection detection and advance sexual health equity among adolescents and young adults.

### Importance

How to implement equitable and efficient sexually transmitted infection screening in the ED remains unclear. Universal testing can reduce stigma but is often impractical in resource-limited settings. Risk-based screening may improve efficiency but risks missing infections.^8–10^ Risk-based computer surveys have shown some promise but offer minimal patient education or shared decisionmaking.^10–13^ Patient decision aids are tools that can facilitate a shared decisionmaking approach and a process in which patients and health care clinicians consider outcome probabilities and patient preferences and reach a mutual agreement.^14^ In other areas of emergency care, decision aids have been developed to overcome these limitations, helping patients and clinicians make informed, value-aligned decisions and improving clinical outcomes.^15–19^ For many adolescents and young adult ED patients, the choice to or not to test for a sexually transmitted infection in the ED may be a reasonable option, particularly when adolescents and young adults have primary care clinicians or have been tested recently. However, no ED-based decision aid currently exists to support sexually transmitted infection testing including at extragenital sites.

### Goals of This Investigation

The objective of this randomized controlled trial was to evaluate the efficacy of a digital patient decision aid, Sexually Transmitted Infection Check in the Emergency Room (STIckER), to increase sexually transmitted infection testing in the ED among sexually active adolescents and young adult patients. Our secondary objective were to assess its acceptability, appropriateness, and feasibility in the ED setting.

## METHODS

### Study Design and Setting

We conducted a randomized controlled trial of health care clinicians in an adult and pediatric urban ED within a large academic medical center in New York City. These sites serve a predominantly Hispanic population, more than 25% of whom live below the federal poverty line. The EDs collectively serve more than 150,000 patients annually and are located in different buildings with separate staffing. In the year prior the study, among 14- to 24-year-old ED patients, rates of any, urinary, rectal, and pharyngeal chlamydia and/or gonorrhea testing were 7.4%, 6.5%, <0.1%, and 0.3%, respectively. The protocol for this study has been previously published.^20^

### Selection and Randomization of Participants

#### Clinicians.

Eligible health care clinicians were attending physicians and physician assistants working ≥5 clinical shifts/month. We recruited health care clinicians via department-wide emails and informational sessions. This included 98 adult ED and 41 pediatric ED attendings as well as 37 physician assistants. Health care clinicians provided informed consent, and we randomized them to either deliver a digital decision aid (intervention) or usual care (control) to adolescents and young adult patients. We performed blocked randomization using the Clinical Trial Randomization Tool (https://ctrandomization.cancer.gov/instructions/).^21^ Intervention arm health care clinicians received virtual training on shared decisionmaking, sexually transmitted infection epidemiology, and the patient decision aid. Control arm health care clinicians received training on sexually transmitted infection epidemiology and shared decisionmaking only. All participating health care clinicians received a $50 incentive.

#### Patients.

English-speaking adolescents and young adult patients aged 14 to 24 years were eligible if they reported sexual activity in the past 6 months. Exclusion criteria included foster care and state custody, as well as cognitive impairment, aggressive behavior, or critical illness, as determined by the attending provider. We received a waiver of parental consent for patients aged 14 to 17 years; all minors of that age range were eligible for screening and participation. Research coordinators screened patients Monday to Friday (8 AM to 10 PM) and during limited weekend hours. Eligible patients assigned to an enrolled health care provider randomized to the intervention arm provided written informed consent, completed a baseline survey, scanned a quick response (QR) code, and completed the STIckER digital tool. Participants assigned to a provider randomized to the control arm also completed a baseline survey and scanned a QR code and received a prompt stating, “If you would like sexually transmitted infection testing today, please tell your provider.” Participants received a $10 incentive. Participants used their personal mobile phones to scan the QR code; if they did not have a mobile device, a tablet was provided for them to use for study purposes. The Columbia University Irving Medical Center Institutional Review Board approved this study, and it was registered on ClinicalTrials.gov (NCT06014177).

### Intervention

The patient decision aid entitled, STIckER, is a web-based app designed to support adolescents and young adult patients in making informed choices about sexually transmitted infection testing during ED visits. We developed the tool through a multiphase process grounded in human-centered design and shared decisionmaking principles and adhered to the International Patient Decision Aid Standards.^22,23^ In the initial development phase, we defined the decision (gonorrhea/chlamydia testing), target population (adolescents and young adult ED patients), and relevant decision options (type and location of sexually transmitted infection testing). A multidisciplinary steering group including emergency physicians, adolescent medicine specialists, public health experts, and adolescents and young adult patients guided the process. We conducted a series of iterative design workshops and alpha testing cycles with adolescents and young adult patients and ED clinicians to refine both the content and the usability of the tool.^24^

**Figure 1.**
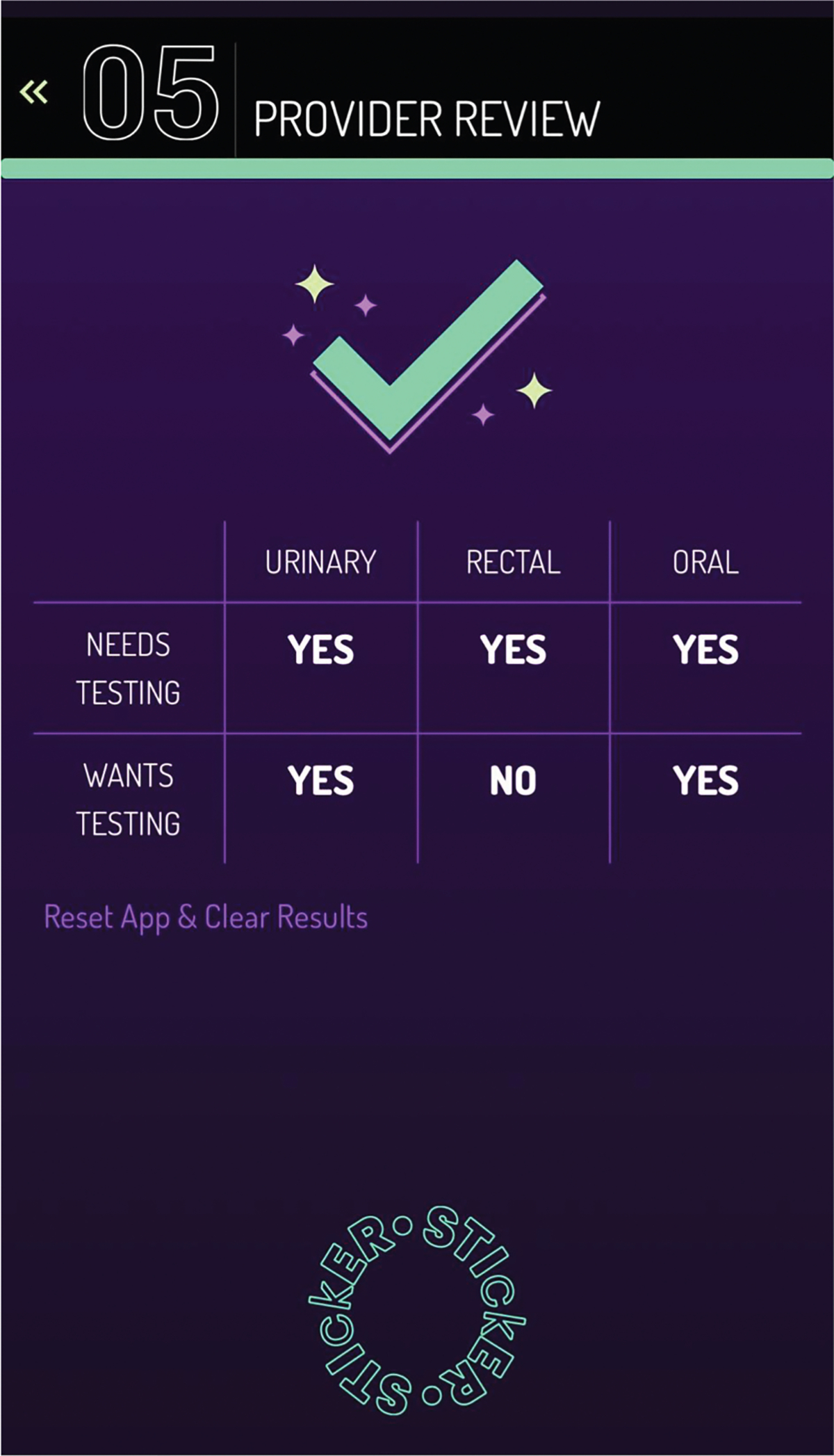
Final infographic.

STIckER includes 5 interactive modules: (1) a brief overview of sexually transmitted infections and the importance of testing, (2) a sexual health risk assessment adapted from validated instruments (eg, *I Want the Kit*) to stratify testing need by anatomical site (oral, rectal, and urinary), (3) personalized screening recommendations, followed by a prompt asking participants whether they want to be screened, (4) a values clarification exercise where participants weigh factors such as privacy and convenience, and (5) a personalized infographic summarizing testing recommendations based on risk and preferences.^24^ As shown in Figure 1, this infographic is designed to be reviewed jointly by the patient and health care provider and to prompt a collaborative discussion about sexually transmitted infection testing recommendations and patient preferences, thereby facilitating in-person shared decisionmaking. We built the app for integration into clinical workflows being delivered to patients before patients have their initial interaction with their ED provider with the infographic discussed during the initial clinical encounter. We designed the app for use on a participant’s personal device (such as a smartphone) via QR code access.

### Outcomes

The primary outcome was the proportion of adolescent and young adult participants who completed any gonorrhea/chlamydia testing during the study visit, as verified by electronic medical record review. The primary analysis compared this proportion between the intervention and control arms. Secondary outcomes included rates of extragenital (ie, oral and rectal) testing, overall and site-specific sexually transmitted infection positivity, and HIV and syphilis testing. Patients self-reported items using validated surveys such as the Shared Decision Making Scale-9 (SDM-9), and the Control Preferences Scale (CPS).^25,26^ Implementation outcomes included the Acceptability of Intervention Measure, Feasibility of Intervention Measure, and Intervention Appropriateness Measure, reported by both adolescents and young adult participants and health care clinicians.^27^

### Data Collection

Adolescents and young adult participants completed 3 surveys: a baseline survey (demographics and medical history), an ED exit survey (testing decisions, decisional conflict, and shared decisionmaking), and intervention-specific implementation scales. All participants completed a sexual health risk assessment that stratified testing needs by anatomical site (oral, rectal, urinary). In the intervention arm, this assessment was embedded within the app, whereas in the control arm it was completed as part of the exit survey. We adopted survey items from a validated tool, a 6-item scale with score ranging from 0 to 10, to measure sexually transmitted infection vulnerability.^11^ Participants with a 0 to 2 score was defined as low need for screening, 3 to 6 as medium need, and 7 to 10 as high need.^12,28^ Together with anatomic exposures in the past 90 days, we further classified the participants into low, medium, and high need of site-specific testing (genitourinary, pharyngeal, and rectal). Health care clinicians completed a baseline demographic survey and a postencounter survey for each adolescents and young adult patient focused on shared decisionmaking and acceptability. Intervention health care clinicians also completed a postimplementation survey covering ease of use, communication skills, and attitudes about STIckER. We managed all data in REDCap, which is a secure web platform for building and managing online databases and surveys.^29^

### Data Analysis

We conducted descriptive statistics to summarize demographic features, self-reported sexual behaviors, sexual health indicators, patient-reported outcomes, health care provider–level outcomes, and implementation measures. We compared proportions for categorical variables (eg, demographic characteristics, sexually transmitted infection testing performed, HIV/syphilis testing, provider sexually transmitted infection testing discussions) as well as mean and SD or median and interquartile range for continuous variables (eg, age, decisional conflict scores, and provider self-efficacy ratings) by study arms. We summarized participants’ sexual health indicators using a composite survey score, and we classified participants into high, medium, or low need for site-specific (genitourinary, rectal, and pharyngeal) sexually transmitted infection testing according to predefined criteria.

We compared proportions for categorical variables (eg, demographic characteristics, sexually transmitted infection testing performed, HIV/syphilis testing, and provider sexually transmitted infection testing discussions) by study arms using chi-square test and Fisher’s exact test if the count was below 5. We compared continuous variables (eg, age, decisional conflict scores, and provider self-efficacy ratings) using Wilcoxon rank sum test. We conducted log-binomial regression with robust standard error to adjust for the provider effect to estimate unadjusted and adjusted risk ratios (aRRs) with 95% confidence intervals (CIs) for the effect of (STIckER intervention versus control on receiving any gonorrhea/chlamydia testing, and site-specific (genitourinary and pharyngeal) testing. Adjusted models accounted for baseline differences by study arm such as prior gonorrhea/chlamydia testing, or other correlates of HIV/sexually transmitted infection testing. We summarized implementation outcomes (Acceptability of Intervention Measure, Feasibility of Intervention Measure, and Intervention Appropriateness Measure) for the subgroup of participants who reported actively using the app to inform their testing decision. We calculated SDM-9 and CPS scores and performed all analyses using R version 4.2.1 (R Core Team) and RStudio 2022.02.03 (Posit Team).

### Power Analysis

Utilizing a simple, parallel randomized design and accounting for clinicians evaluating more than one patient, we estimated the minimum detectable effect size of 0.348. We based the minimal detectable effect size on the following assumptions: 0.05 alpha (*α*), a two-tailed test, 80% power, and intraclass correlation coefficient of 0.01, 50% of variance in adolescents and young adults and 50% of variance in clinicians explained, and we balanced all other covariates of interest (eg, age) by randomization of STIckER to clinicians and their adolescents and young adult patients. With those assumptions, a sample size of 44 clinicians and 140 adolescents and young adults would have had the power to detect a minimum of 35% higher likelihood of being tested for sexually transmitted infections in the intervention arm compared to usual care.

## RESULTS

We consented and randomized 44 ED health care clinicians, 21 in the pediatric ED and 23 in the adult ED. One health care clinician withdrew due to medical leave, and three did not complete the poststudy survey. Although we enrolled more physician assistants from the adult ED and more general pediatricians from the pediatric ED, we found minimal differences in clinician groups by study arm and health care clinicians reported similar years of clinical experience (Table E1, available at http://www.annemergmed.com). Health care clinicians in the intervention and control arm enrolled a median of 3 (interquartile range 2 to 4) and 3 (interquartile range 2 to 5) adolescents and young adult patients, respectively.

**Figure 2.**
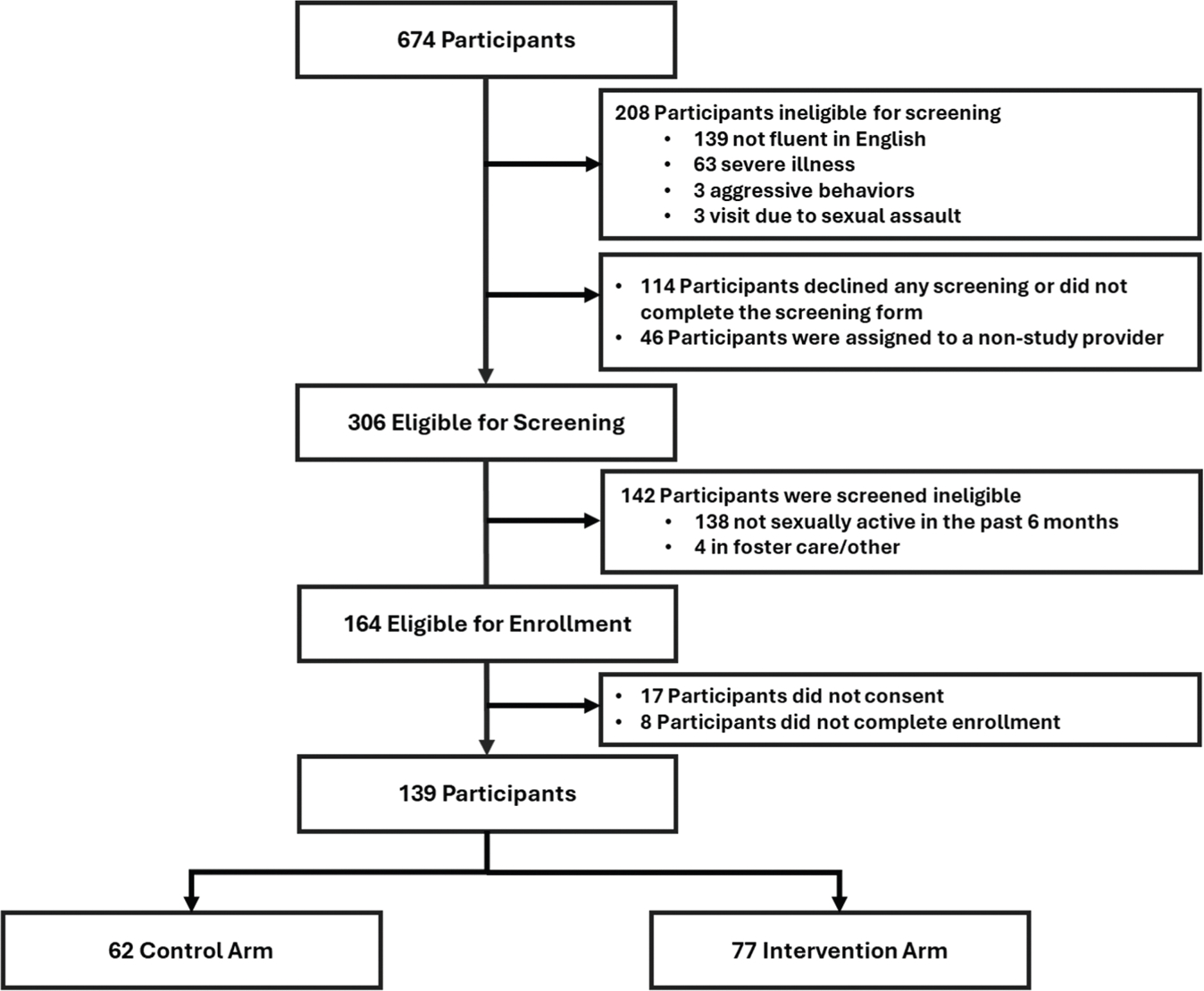
Consolidated Standards of Reporting Trials diagram. (A flow diagram illustrating progress leading to the analytic cohort of intervention and control arms.)

From June 2023 to June 2024, we enrolled 139 out of the 164 eligible adolescent and young adult participants (84.8%), 62 in the control arm and 77 in the STIckER intervention arm (Figure 2). Baseline demographic characteristics were similar between arms as seen in Table 1. The median age was 19 years (intraquartile range, 18 to 22) in the intervention arm and 20 years (interquartile range, 19 to 22) in the control arm. Most participants were assigned female at birth, identified as female, identified as heterosexual, and self-identified as Hispanic. Nearly 70% reported having seen a clinician for a routine physical exam in the past year. The distribution of encounters presenting with symptoms commonly associated with sexually transmitted infection evaluation (eg, concern for an sexually transmitted infection, dysuria, pharyngitis, and abdominal pain) was similar across arms. Among these, a higher proportion of control participants reported HIV testing (42% vs 19%; 95% CI, 5% to 33%) and sexually transmitted infection testing (56% vs 37%; 95% CI, 2% to 33%) compared with intervention participants; these 2 variables were different between arms and therefore we adjusted for them in the regression analysis.

Sexual health behaviors did not differ between arms (Table E2, available at http://www.annemergmed.com). Few participants reported always using condoms, whereas most reported occasional or no condom use. Patterns of sexual activity were also comparable, with most participants reporting oral sex and vaginal sex, and the majority reporting no anal sex.

Sexual health assessments used to classify sexually transmitted infection testing needs also did not differ between arms (Table E3, available at http://www.annemergmed.com). In the intervention arm, 81% were classified as having a high need for genitourinary testing, compared with 90% in the control arm (95% CI, −21% to 2%). For pharyngeal testing, we categorized more intervention participants as high or medium need (68% vs 54%; 95% CI, −3% to 30%; 11% vs 7%; 95% CI, −5% to 14%) and fewer as low need (21% vs 39%) compared with controls (95% CI, −34% to −3%).

### Primary and Secondary Clinical Outcomes

The STIckER app was associated with higher rates of sexually transmitted infection testing in the ED. Gonorrhea/chlamydia testing was performed more often in the intervention arm than in the control arm (45% vs 26%; 95% CI, 2% to 36%) (Table 2). Genitourinary (43% vs 24%; 95% CI, 2% to 36%) and pharyngeal testing (16% vs 3%; 95% CI, 2% to 23%) were also more frequently completed in the intervention arm. HIV and syphilis testing rates did not differ. In log-binomial analyses, participants randomized to the intervention were 1.73 times more likely to receive gonorrhea/chlamydia testing (95% CI, 1.09 to 2.94), 1.79 times more likely to receive genitourinary testing (95% CI, 1.11 to 3.11), and 4.89 times more likely to receive pharyngeal testing (95% CI, 1.40 to 30.71). Associations remained after adjustment for prior HIV/sexually transmitted infection testing in the past year (gonorrhea/chlamydia: aRR, 1.76; 95% CI, 1.10 to 3.00; genitourinary: aRR, 1.84; 95% CI, 1.13 to 3.20; pharyngeal: aRR, 4.56; 95% CI, 1.30 to 28.66). Genitourinary testing revealed positivity for 2 chlamydia and 3 gonorrhea cases, all of which were in the intervention arm. All other tests were negative. Twelve patients received both pharyngeal and genitourinary testing.

### Patient-Reported Outcomes

Patient-reported outcomes differed by randomized arm (Table E4, available at http://www.annemergmed.com). More participants randomized to the intervention arm reported that they discussed sexually transmitted infection testing with their health care clinicians (75% vs 41%; 95% CI, 23% to 54%) and rated the information received about sexually transmitted infections as more comprehensive, clear, and helpful. They also reported slightly higher overall ratings of ED care (median, 9 vs 8; 95% CI, −0.04 to 2). Several decisional conflict items similarly favored the intervention, including greater clarity on the need for sexually transmitted infection testing, understanding of available options, and thorough weighing of options. However, the SDM-9 score and decisionmaking role defined by the CPS did not differ by arms. Both arms reported that they took an active role in deciding whether to get tested for sexually transmitted infections, either by making the decision on their own or after considering the information from the app or the provider.

### Provider Outcomes

Provider-reported outcomes also differed between groups (Table E5, available at http://www.annemergmed.com). Patient–provider discussions of sexually transmitted infection testing occurred more frequently in the intervention arm, with a greater proportion of patients initiating the discussion (24% vs 5%; 95% CI, 9% to 31%) and fewer encounters in which testing was not discussed (33% vs 54%; 95% CI, −36% to 3%). Clinicians in the intervention arm were also more likely to order sexually transmitted infection testing (53% vs 28%; 95% CI, 7% to 42%), but perception on how this decision was made did not differ between arms.

### Implementation Outcomes

Participants in the STIckER arm who spoke to their health care provider about sexually transmitted infection testing (n=60) reported high acceptability, appropriateness, and feasibility, as evidenced by an acceptability score of 18 (IQR 16, 20), appropriateness score of 16 (IQR 16, 20) and feasility score of 16 (IQR 16, 20) (Table E6, available at http://www.annemergmed.com). Each measure ranges from 4 to 20, with higher scores indicating greater perceived acceptability, appropriateness, and feasibility.

Intervention health care clinicians used the app often to discuss sexually transmitted infection testing (63%; 51 of 76). Of those STIckER intervention clinician-patient interactions where sexually transmitted infection testing was discussed (n=51), we collected implementation outcomes on the clinician exit surveys. The tool demonstrated high acceptability, appropriateness, and feasibility, as evidenced by an acceptability score of 5 (IQR 4, 5) (one question, max 5), appropriateness score of 15 (IQR 12, 15) (3 questions asked, max 15), and feasibility score of 20 (IQR 16, 20) (4 questions asked, max 20) (Table E7, available at http://www.annemergmed.com).

Provider self-efficacy in conducting sexually transmitted infection–related conversations was lower among those randomized to the intervention arm compared with the control arm (Table E8, available at http://www.annemergmed.com). Intervention clinicians reported less confidence in structuring sexually transmitted infection conversations, demonstrating appropriate nonverbal behavior, and checking patient understanding.

### LIMITATIONS

We conducted the study at a single urban academic medical center, which may limit generalizability to other ED settings, particularly those with fewer resources or different patient demographics. Participants were predominantly Hispanic and female identifying, reflecting the local population but limiting generalizability to other groups. We did not collect a large enough sample size to adequately power to stratify by need for sexually transmitted infection testing nor collect the data to compare enrolled to unenrolled participants. Also, limiting the study to English-speaking participants only also limited its generalizability. Self-reported sexual behaviors may be influenced by social desirability bias, though we verified primary outcomes through electronic medical review. Although randomization balanced most baseline characteristics, residual confounding is possible, including from differences in prior sexually transmitted infection testing history. Randomization at the provider level minimized contamination but introduced clustering that may have reduced statistical efficiency. Also, we did not collect data on why adolescents and young adults in the intervention did not interact with the app; this would have added to our acceptability outcomes. Finally, we did not evaluate linkage to follow-up care for those testing positive, an important next step for sustaining sexually transmitted infection prevention efforts.

## DISCUSSION

In this randomized trial of a digital patient decision aid for sexually transmitted infection testing among adolescents and young adults presenting to the ED, we found that use of the decision aid increased the proportion of patients who completed gonorrhea/chlamydia testing. The effect was consistent across both genitourinary and extragenital testing, with nearly 5-fold higher likelihood of pharyngeal testing among intervention participants. Importantly, patients in the intervention arm also reported greater scores in shared decisionmaking, including higher engagement in discussions with clinicians, clearer understanding of testing options, and higher ratings of information clarity and helpfulness. These findings highlight the potential of structured, patient-facing tools to enhance sexual health decisionmaking in acute care settings.

We can attribute STIckER’s success to several factors. To start, STIckER incorporates multiple mechanisms to encourage engagement in sexual health care. The app provided personalized, pre-encounter information about individual testing needs and options, shifting patients from passive recipients to active participants in decisionmaking.^30,31^ Visual infographics summarizing risk and testing recommendations aimed to empower patients to initiate conversations about sexually transmitted infection testing. The app’s structured modular design clarified options and sought to align testing choices with patient values; these elements of decisionmaking conversations are often absent when emergency care needs to focus on acute issues. This clarity likely contributed to the increase in overall and pharyngeal sexually transmitted infection testing seen in this trial, a clinically meaningful outcome given that extragenital infections are common and often missed without targeted screening.

Another likely key contributor to STIckER’s impact was its development through human-centered design.^32^ Rather than adapting an existing tool to the ED, we created STIckER with direct input from adolescent and young adult patients who pilot tested the app during their ED visits.^24^ Through iterative design workshops and usability testing, we refined not only the app’s content but also its tone, flow, and visual layout. This participatory process ensured that the tool was developmentally appropriate for adolescents, intuitive to use in a stressful ED environment, and responsive to user needs. STIckER also addressed barriers that traditional provider-led screening may overlook, such as uncertainty about what tests are offered or how they are performed.^33^ By normalizing sensitive sexual health discussions and minimizing stigma, the app encouraged patient action while adding minimal burden for clinicians, which is essential for maintaining patient flow in the ED. These findings underscore how decision aids developed with human-centered design principles can enhance both testing rates and the quality of patient–provider communication.

Beyond its clinical impact, STIckER demonstrated strong potential for real-world adoption. We built the tool to fit within ED workflows, requiring no additional staffing and no electronic medical record integration. As a web-based app accessed by QR code, STIckER avoided installation barriers and functioned on patients’ personal devices, enhancing scalability.^34^ Both adolescents and clinicians rated the tool as highly acceptable, appropriate, and feasible, with especially high ratings among participants who used it to guide testing decisions. Importantly, STIckER performed well across two distinct clinical environments, adult and pediatric EDs, suggesting adaptability across varied staffing models and cultures. This ease of implementation underscores STIckER’s potential to overcome long-standing structural barriers to preventive sexual health services in the ED.

An unexpected finding was the lower self-efficacy reported by clinicians in the intervention arm when discussing sexually transmitted infection testing. Rather than signaling diminished competence, this may reflect the recalibration of roles that occurs when decisionmaking becomes more patient-centered. As patients arrived informed and ready to engage, clinicians may have felt less control over the encounter or less certain about how to respond to patient-initiated requests. This tension may occur when new models of shared decisionmaking are introduced and reveal important gaps in provider preparation rather than resistance. Targeted sexually transmitted infection education, communication training, and scripting tools could help align provider confidence with patient activation.

Another important finding was the limited uptake of rectal, HIV, and syphilis testing despite many participants reporting behaviors indicating a need for these tests. This disconnect between identified risk and completed testing suggests that awareness alone, whether among patients or clinicians, is insufficient to ensure action. Although STIckER focused on gonorrhea/chlamydia, the persistent underuse of HIV and syphilis testing highlights an opportunity for future versions to incorporate broader sexual health decision support. Prior studies show that provider education can temporarily increase testing rates, but these gains often fade without systems-level supports.^33^ Persistent barriers may include patient reluctance to request certain tests, provider discomfort or uncertainty about ordering them, and logistical challenges such as unclear responsibility for specimen collection. Rectal testing may be particularly challenging because ED clinicians commonly perform throat swabs but are less accustomed to collecting rectal specimens. Addressing structural and cultural barriers through team-based protocols, embedded order sets, and automated prompts that ensure comprehensive testing is critical.^35^ Given the high prevalence of asymptomatic infections among adolescents and young adults, closing this gap remains essential for any ED-based sexually transmitted infection prevention strategy.

In summary, implementation of the STIckER app in the ED increased rates of gonorrhea/chlamydia testing, particularly at extragenital sites, and improved patient engagement in sexual health decisionmaking. The tool was highly acceptable to patients, feasible for integration into ED workflows, and required minimal provider time. These findings demonstrate that structured, patient-centered digital tools can strengthen sexual health care delivery in acute care settings. Future studies should evaluate STIckER in diverse ED environments, test its integration with clinical systems, and expand its application to include HIV, syphilis, and potentially sexually transmitted infection prevention (HIV postexposure prophylaxis and preexposure prophylaxis and doxycycline preexposure prophylaxis) decision support. As health systems seek scalable strategies to close sexually transmitted infection screening gaps, STIckER offers a model for how participatory design, behavioral science, and digital innovation can advance patient-driven care in even the most time-pressured clinical environments.

## Supplementary Material

MMC1

## Figures and Tables

**Table 1.** Demographics of study participants.

	Control	Intervention
Characteristic	(n = 62)	(n = 77)

Patient age, median (IQR)	20 (19–22)	19 (18–22)
**Sex at birth, n (%)**		
Male	19 (30.6)	24 (31.2)
Female	43 (69.4)	53 (68.8)
**Gender identity, n (%)**		
Male	18 (29.0)	24 (31.2)
Female	41 (66.1)	52 (67.5)
Nonbinary/transgender	3 (4.8)	1 (1.3)
**Sexual orientation, n (%)**		
Bisexual	10 (16.1)	10 (13.0)
Gay	3 (4.8)	2 (2.6)
Heterosexual	43 (69.4)	62 (80.5)
I am not sure about my sexual identity	0 (0)	1 (1.3)
I describe my sexual identity some other way	1 (1.6)	2 (2.6)
Pansexual	5 (8.1)	0 (0)
**Race/ethnicity, n (%)**		
Asian	1 (1.6)	2 (2.6)
Black or African American	7 (11.3)	11 (14.3)
Hispanic or Latino	51 (82.3)	62 (80.5)
Non-Hispanic White	3 (4.8)	2 (2.6)
**Over the past year, did you see a doctor or nurse for a physical examination when you were not sick?, n (%)**		
Yes	43 (69.4)	53 (68.8)
No	16 (25.8)	23 (29.9)
Unsure	3 (4.8)	1 (1.3)
**Service received, n (%)** *		
Information on birth control	20 (42)	21 (39)
HIV testing	20 (42)	10 (19)
Testing for sexually transmitted infections	27 (56)	20 (37)
Other	12 (25)	18 (33)

*IQR,* Interquartile range.

* Of the 120 who saw a doctor/nurse in the past year.

**Table 2.** Primary and secondary testing efficacy outcomes around sexually transmitted infection testing.

Characteristic	Control (n = 62)	Intervention (n = 76)	Unadjusted Risk Ratio	Unadjusted 95% CI	Adjusted Risk Ratio	Adjusted 95% CI

**Primary outcome**						
Any gonorrhea/chlamydia testing	16 (26%)	34 (45%)	1.73	1.09–2.94	1.76	**1.10–3.00**
**Secondary outcome**						
Genitourinary testing	15 (24%)	33 (43%)	1.79	1.11–3.11	1.84	**1.13–3.20**
Pharyngeal testing	2 (3.1%)	12 (16%)	4.89	1.40–30.71	4.56	**1.30–28.66**
HIV testing	6 (9.7%)	11 (14%)	1.50	0.60–4.13	1.49	0.59–4.15
Syphilis testing	5 (8.1%)	10 (13%)	1.63	0.61–5.01	1.60	0.60–4.97

Bolded confidence intervals are statistically significant.

## Data Availability

A partial or complete data set is available upon reasonable request to the corresponding author to investigators who provide an institutional review board letter of approval.

## Editor’s Capsule Summary

### What is already known on this topic

Some adolescents and young adults have sexually transmitted infections that go undetected during emergency department (ED) visits.

### What question this study addressed

Does a patient-facing tablet tool increase sexually transmitted infection testing in the ED?

### What this study adds to our knowledge

Patients randomized to the intervention arm (n = 76) were more likely than those in the control arm (n = 62) to receive any gonorrhea/chlamydia testing (45% versus 26%; difference 19%; 95% confidence interval 2% to 36%).

### How this is relevant to clinical practice

Digital patient decision aids could help improve sexually transmitted infection detection in the ED.
