# Supplementary material for: A Randomized Controlled Trial of a Digital Patient Decision Tool to Increase Sexually Transmitted Infection Testing in the Emergency Department
Source: Ann Emerg Med. Author manuscript; Available in PMC 2026 Jun 30. (PMC13313571; doi:10.1016/j.annemergmed.2026.03.016)
Supplement: MMC1 [file NIHMS2177262-supplement-MMC1.docx]

**SUPPLEMENTAL TABLES**

**Supplemental Table E1. Demographic Features of Healthcare Providers (N=41)**

| **Characteristic** | **Control (N = 20)** | **Intervention (N = 21)** |
| --- | --- | --- |
| **Provider age in years**, median (IQR) | 36 (32, 43) | 36 (32, 40) |
| **Sex at birth**, n (%) |  |  |
| Male | 6 (30) | 7 (33) |
| Female | 14 (70) | 14 (67) |
| **Race and Ethnicity**, n (%) |  |  |
| Asian | 3 (15) | 5 (24) |
| Black or African American | <5 | <5 |
| Hispanic or Latino | <5 | <5 |
| White | 14 (70) | 12 (57) |
| Other | <5 | <5 |
| **Years working in an ED since completion of medical training**, median (IQR) | 5 (3, 12) | 5 (2, 10) |
| **What is your most advanced clinical training?** n (%) |  |  |
| Emergency Medicine | 9 (45) | 7 (33) |
| General Pediatrics | <5 | 5 (24) |
| Pediatric Emergency Medicine | 7 (35) | 5 (24) |
| Physician Assistant | <5 | <5 |

**Three HCPs did not complete the demographic study. IQR= interquartile range.*

**Supplemental Table E2. Sexual Health Behaviors (N=133*)**

| **Characteristic** | **Overall (N = 133*)** | **Control (N = 61)** | **Intervention (N = 72)** |
| --- | --- | --- | --- |
| **Age, years**, median (IQR) | 20 (18, 22) | 20 (19, 22) | 19 (18, 22) |
| **New or multiple partners in the last 90 days**, n (%) | 46 (34) | 18 (29) | 28 (39) |
| **>1 current sex partner**, n (%) | 12 (9.0) | 6 (9.7) | 6 (8.3) |
| **History of STI diagnosis/treatment**, n (%) | 36 (27) | 16 (26) | 20 (28) |
| **Number of sex partners in the past 90 days**, n (%) |  |  |  |
| 0-1 | 103 (78) | 48 (79) | 55 (76) |
| 2-4 | 29 (22) | 12 (19) | 17 (24) |
| 5-9 | 0 (0) | 0 (0) | 0 (0) |
| 10+ | 1 (0.7) | 1 (1.6) | 0 (0) |
| **Condom use during sex**, n (%) |  |  |  |
| Always | 19 (14) | 9 (15) | 10 (14) |
| Sometimes | 59 (44) | 27 (44) | 32 (44) |
| No | 55 (41) | 25 (41) | 30 (42) |
| **Who you had sex with**, n (%) |  |  |  |
| Partner-w-Penis | 80 (60) | 38 (62) | 42 (58) |
| Partner-w-Penis, Partner-w-Vagina | 16 (12) | 8 (13) | 8 (11) |
| Partner-w-Vagina | 37 (28) | 15 (25) | 22 (31) |
| **Oral sex type in the past 3 months**, n (%) |  |  |  |
| Received | 19 (14) | 10 (16) | 9 (12) |
| Performed | 12 (9) | 5 (8.2) | 7 (9.7) |
| Received and performed | 80 (60) | 35 (57) | 45 (62) |
| No | 22 (17) | 11 (18) | 11 (15) |
| **Anal sex type in the past 3 months**, n (%) |  |  |  |
| Received | 9 (6.8) | 5 (8.2) | 4 (5.6) |
| Performed | 7 (5.3) | 2 (3.3) | 5 (6.9) |
| Received and performed | 2 (1.5) | 0 (0) | 2 (2.8) |
| No | 115 (86) | 54 (89) | 61 (85) |
| **Vaginal sex type in the past 3 months**, n (%) |  |  |  |
| Received | 80 (60) | 37 (61) | 43 (60) |
| Performed | 30 (23) | 13 (21) | 17 (24) |
| No | 23 (17) | 11 (18) | 12 (17) |
| **Tested for GC/CT in the past month**, n (%) |  |  |  |
| Yes | 32 (24) | 17 (28) | 15 (21) |
| No | 93 (70) | 40 (66) | 53 (74) |
| Unsure | 8 (6) | 4 (6.6) | 4 (5.6) |

**133 represents the number of participants who completed the behavioral survey. IQR= interquartile range.*

**Supplemental Table E3. Sexual health assessment scores between arms and testing recommendations**

| **Characteristic** | **Overall (N = 133*)** | **Control (N = 61)** | **Intervention (N = 72)** |
| --- | --- | --- | --- |
| **Overall sexual health survey score**, median (IQR) | 4 (4, 5) | 4 (4, 5) | 4 (4, 6) |
| **Need for STI Testing**, n (%) |  |  |  |
| High need | 118 (89) | 55 (90) | 63 (88) |
| Medium need | 10 (7.5) | 5 (8.2) | 5 (6.9) |
| Low need | 5 (3.8) | 1 (1.6) | 4 (5.6) |
| **Genitourinary**, n (%) |  |  |  |
| High need | 113 (85) | 55 (90) | 58 (81) |
| Medium need | 10 (7.5) | 5 (8.2) | 5 (6.9) |
| Low need | 10 (7.5) | 1 (1.6) | 9 (12) |
| **Pharyngeal**, n (%) |  |  |  |
| High need | 82 (62) | 33 (54) | 49 (68) |
| Medium need | 12 (9) | 4 (6.6) | 8 (11) |
| Low need | 39 (29) | 24 (39) | 15 (21) |
| **Rectal**, n (%) |  |  |  |
| High need | 10 (7.5) | 4 (6.6) | 6 (8.3) |
| Medium need | 18 (14) | 6 (9.8) | 12 (17) |
| Low need | 105 (79) | 51 (84) | 54 (75) |

**133 represents the number of participants who completed the behavioral survey.*

**Supplemental Table E4: Secondary patient-based efficacy outcomes**

| **Characteristic** | **Overall (N=137*)** | **Control (N = 61)** | **Intervention (N = 72)** |
| --- | --- | --- | --- |
| **Did you discuss STI testing with your medical provider during your ER visit today?** n (%) |  |  |  |
| Yes | 82 (60) | 25 (41) | 57 (75) |
| No | 51 (37) | 35 (57) | 16 (21) |
| Can't remember | 4 (2.9) | 1 (1.6) | 3 (3.9) |
| **Of those discussing or could not remember discussing STI Testing….** | | | |
|  | **Overall (N = 86)** | **Control (N = 26)** | **Intervention (N = 60)** |
| **Which of the following options best describes your desire regarding STI testing today?** n (%) |  |  |  |
| Want to be tested today | 41 (48) | 9 (35) | 32 (53) |
| Did not want to be tested today | 18 (21) | 4 (15) | 14 (23) |
| Neutral on wanting | 26 (30) | 13 (50) | 13 (22) |
| Don't remember | 1 (1.2) | 0 (0) | 1 (1.7) |
| **How was this decision made**, n (%) |  |  |  |
| I made this decision my own | 56 (66) | 18 (69) | 38 (64) |
| I made the decision after seriously considering the medical provider’s opinion//// after considering the information in the STIckER app and my medical provider | 18 (21) | 4 (15) | 14 (24) |
| The medical provider and I shared the responsibility for making the decision after considering our opinions | 7 (8.2) | 2 (7.7) | 5 (8.5) |
| The medical provider made the decision after seriously considering my opinion | 2 (2.4) | 0 (0) | 2 (3.4) |
| The medical provider made the decision on their own | 2 (2.4) | 2 (7.7) | 0 (0) |
| **How would you describe the amount of information you received about STIs and testing choices during this visit?*** median (IQR) | 5 (3, 5) | 3 (3, 3) | 5 (5, 5) |
| **How clear was the information about STIs and testing choices?*** median (IQR) | 7 (5, 10) | 5 (3, 6) | 9 (7, 10) |
| **How helpful was the information?*** median (IQR) | 7 (5, 9) | 6 (3, 6) | 9 (7, 10) |
| **What number would you use to rate your care during this emergency room visit?*** median (IQR) | 9 (7, 10) | 8 (6, 10) | 9 (8, 10) |
| **Would you recommend the way that you and your medical provider shared information about STIs to other patients?*** median (IQR) | 9 (7, 10) | 9 (7, 10) | 9 (6.5, 10) |
| **Would you recommend this emergency room to your friends and family?** n (%) |  |  |  |
| Definitely no | 0 (0) | 0 (0) | 0 (0) |
| Probably no | 3 (3.5) | 3 (12) | 0 (0) |
| Probably yes | 29 (34) | 8 (31) | 21 (35) |
| Definitely yes | 54 (63) | 15 (58) | 39 (65) |
| **9-item Shared Decision-Making Questionnaire (SDM9)** | | | |
| **Shared Decision-Making Questionnaire (SDM-Q-9)**, median (IQR) | 80 (67, 98) | 83 (64, 98) | 80 (72, 96) |
| **It was clear to me that a decision regarding STI testing needs to be made**, n (%) |  |  |  |
| Completely disagree | 0 (0) | 0 (0) | 0 (0) |
| Strongly disagree | 3 (3.5) | 0 (0) | 3 (5) |
| Somewhat disagree | 2 (2.3) | 1 (3.8) | 1 (1.7) |
| Somewhat agree | 13 (15) | 8 (31) | 5 (8.3) |
| Strongly agree | 28 (33) | 2 (7.7) | 26 (43) |
| Completely agree | 40 (47) | 15 (58) | 25 (42) |
| **It was clear to me how I could be involved in making the decision**, n (%) |  |  |  |
| Completely disagree | 1 (1.2) | 0 (0) | 1 (1.7) |
| Strongly disagree | 2 (2.3) | 0 (0) | 2 (3.3) |
| Somewhat disagree | 1 (1.2) | 1 (3.8) | 0 (0) |
| Somewhat agree | 12 (14) | 4 (15) | 8 (13) |
| Strongly agree | 28 (33) | 3 (12) | 25 (42) |
| Completely agree | 42 (49) | 18 (69) | 24 (40) |
| **I was told that there are different options for testing**, n (%) |  |  |  |
| Completely disagree | 4 (4.7) | 2 (7.7) | 2 (3.3) |
| Strongly disagree | 4 (4.7) | 1 (3.8) | 3 (5) |
| Somewhat disagree | 5 (5.8) | 5 (19) | 0 (0) |
| Somewhat agree | 9 (10) | 2 (7.7) | 7 (12) |
| Strongly agree | 26 (30) | 2 (7.7) | 24 (40) |
| Completely agree | 38 (44) | 14 (54) | 24 (40) |
| **I was precisely explained the advantages and disadvantages of STI testing**, n (%) |  |  |  |
| Completely disagree | 4 (4.7) | 2 (7.7) | 2 (3.3) |
| Strongly disagree | 4 (4.7) | 1 (3.8) | 3 (5) |
| Somewhat disagree | 3 (3.5) | 0 (0) | 3 (5) |
| Somewhat agree | 19 (22) | 9 (35) | 10 (17) |
| Strongly agree | 23 (27) | 1 (3.8) | 22 (37) |
| Completely agree | 33 (38) | 13 (50) | 20 (33) |
| **I was helped to understand all the information**, n (%) |  |  |  |
| Completely disagree | 2 (2.3) | 2 (7.7) | 0 (0) |
| Strongly disagree | 2 (2.3) | 1 (3.8) | 1 (1.7) |
| Somewhat disagree | 3 (3.5) | 1 (3.8) | 2 (3.3) |
| Somewhat agree | 15 (17) | 5 (19) | 10 (17) |
| Strongly agree | 22 (26) | 2 (7.7) | 20 (33) |
| Completely agree | 42 (49) | 15 (58) | 27 (45) |
| **I was asked which testing option I prefer**, n (%) |  |  |  |
| Completely disagree | 4 (4.7) | 3 (12) | 1 (1.7) |
| Strongly disagree | 4 (4.7) | 1 (3.8) | 3 (5) |
| Somewhat disagree | 6 (7) | 3 (12) | 3 (5) |
| Somewhat agree | 13 (15) | 4 (15) | 9 (15) |
| Strongly agree | 22 (26) | 4 (15) | 18 (30) |
| Completely agree | 37 (43) | 11 (42) | 26 (43) |
| **I thoroughly weighed the different testing options**, n (%) |  |  |  |
| Completely disagree | 5 (5.8) | 5 (19) | 0 (0) |
| Strongly disagree | 1 (1.2) | 0 (0) | 1 (1.7) |
| Somewhat disagree | 5 (5.8) | 0 (0) | 5 (8.3) |
| Somewhat agree | 15 (17) | 6 (23) | 9 (15) |
| Strongly agree | 21 (24) | 3 (12) | 18 (30) |
| Completely agree | 39 (45) | 12 (46) | 27 (45) |
| **Using the STIckER app, my doctor and I selected a testing option together**, n (%) |  |  |  |
| Completely disagree | 8 (9.3) | 7 (27) | 1 (1.7) |
| Strongly disagree | 4 (4.7) | 0 (0) | 4 (6.7) |
| Somewhat disagree | 6 (7) | 0 (0) | 6 (10) |
| Somewhat agree | 17 (20) | 4 (15) | 13 (22) |
| Strongly agree | 20 (23) | 7 (27) | 13 (22) |
| Completely agree | 31 (36) | 8 (31) | 23 (38) |
| **Using the STIckER app, my doctor and I reached an agreement on how to proceed**, n (%) |  |  |  |
| Completely disagree | 5 (5.8) | 4 (15) | 1 (1.7) |
| Strongly disagree | 2 (2.3) | 1 (3.8) | 1 (1.7) |
| Somewhat disagree | 2 (2.3) | 0 (0) | 2 (3.3) |
| Somewhat agree | 17 (20) | 4 (15) | 13 (22) |
| Strongly agree | 23 (27) | 5 (19) | 18 (30) |
| Completely agree | 37 (43) | 12 (46) | 25 (42) |

**This question was on a scale of 10 being the highest or most to 0 being the lowest or least.*

**Supplemental Table E5: Provider-Based Secondary Outcomes**

| **Characteristic** | **Overall (N = 137)** | **Control (N = 61)** | **Intervention (N = 72)** |
| --- | --- | --- | --- |
| **Did you or your patient discuss STI testing?** n (%) |  |  |  |
| Yes, I brought it up | 58 (42) | 25 (41) | 33 (43) |
| Yes, the patient brought it up | 21 (15) | 3 (4.9) | 18 (24) |
| No neither of us brought it up | 58 (42) | 33 (54) | 25 (33) |
| **STI testing was ordered on your enrolled patient today,** n (%) | 57 (42) | 17 (28) | 40 (53) |
| **How was this decision made?** n (%) |  |  |  |
| On my own | 12 (21) | 6 (33) | 6 (15) |
| After considering the values and preferences of the patient | 6 (10) | 1 (5.6) | 5 (12) |
| The patient and I shared the responsibility | 23 (40) | 8 (44) | 15 (38) |
| The patient made the decision after seriously considering my opinion | 5 (8.6) | 2 (11) | 3 (7.5) |
| The patient made the decision on their own | 12 (21) | 1 (5.6) | 11 (28) |

**137 represents the number of post-exit surveys completed of the 139 encounters.*

**Supplemental Table E6: Patient-based Implementation Outcomes (n=60).**

| **Acceptability of Intervention Measure (AIM)**, median (IQR) | **18 (16, 20)** |
| --- | --- |
| **The STIckER app meets my approval**, n (%) |  |
| Completely disagree | 0 (0) |
| Disagree | 1 (1.7) |
| Neutral | 2 (3.3) |
| Agree | 24 (40) |
| Completely agree | 33 (55) |
| **The STIckER app is appealing to me**, n (%) |  |
| Completely disagree | 0 (0) |
| Disagree | 1 (1.7) |
| Neutral | 4 (6.7) |
| Agree | 28 (47) |
| Completely agree | 27 (45) |
| **I like the STIckER app**, n (%) |  |
| Completely disagree | 0 (0) |
| Disagree | 0 (0) |
| Neutral | 4 (6.7) |
| Agree | 27 (45) |
| Completely agree | 29 (48) |
| **I welcome the STIckER app**, n (%) |  |
| Completely disagree | 0 (0) |
| Disagree | 0 (0) |
| Neutral | 2 (3.3) |
| Agree | 30 (50) |
| Completely agree | 28 (47) |
| **Intervention Appropriateness Measure (IAM)** | **16 (16, 20)** |
| **The STIckER app seems fitting in the Emergency Department**, n (%) |  |
| Completely disagree | 0 (0) |
| Disagree | 0 (0) |
| Neutral | 5 (8.6) |
| Agree | 27 (47) |
| Completely agree | 26 (45) |
| **The STIckER app seems suitable in the Emergency Department**, n (%) |  |
| Completely disagree | 0 (0) |
| Disagree | 2 (3.4) |
| Neutral | 3 (5.2) |
| Agree | 27 (47) |
| Completely agree | 26 (45) |
| **The STIckER app seems applicable in the Emergency Department**, n (%) |  |
| Completely disagree | 0 (0) |
| Disagree | 1 (1.7) |
| Neutral | 4 (6.9) |
| Agree | 29 (50) |
| Completely agree | 24 (41) |
| **The STIckER app seems like a good match in the Emergency Department**, n (%) |  |
| Completely disagree | 0 (0) |
| Disagree | 1 (1.7) |
| Neutral | 5 (8.6) |
| Agree | 26 (45) |
| Completely agree | 26 (45) |
| **Feasibility of Intervention Measure (FIM)** | **16 (16, 20)** |
| **The STIckER app seems implementable in the Emergency Department**, n (%) |  |
| Completely disagree | 0 (0) |
| Disagree | 3 (5.2) |
| Neutral | 5 (8.6) |
| Agree | 27 (47) |
| Completely agree | 23 (40) |
| **The STIckER app seems possible in the Emergency Department**, n (%) |  |
| Completely disagree | 0 (0) |
| Disagree | 2 (3.4) |
| Neutral | 2 (3.4) |
| Agree | 30 (52) |
| Completely agree | 24 (41) |
| **The STIckER app seems doable in the Emergency Department**, n (%) |  |
| Completely disagree | 0 (0) |
| Disagree | 2 (3.4) |
| Neutral | 2 (3.4) |
| Agree | 31 (53) |
| Completely agree | 23 (40) |
| **The STIckER app seems easy to use in the Emergency Department**, n (%) |  |
| Completely disagree | 0 (0) |
| Disagree | 1 (1.8) |
| Neutral | 3 (5.3) |
| Agree | 24 (42) |
| Completely agree | 29 (51) |

**n=60 of those patient encounters that discussed STI testing*

**Supplemental table E7: Intervention Provider-based Implementation Outcomes (n=51)**

| **Acceptability of Intervention Measure (AIM)**, median (IQR) | 5 (4,5) |
| --- | --- |
| **The STIckER app meets my approval**, n (%) |  |
| Completely disagree | 0 (0) |
| Disagree | 0 (0) |
| Neutral | 3 (5.9) |
| Agree | 21 (41) |
| Completely agree | 27 (53) |
| **Intervention Appropriateness Measure (IAM)**, median (IQR) | 15 (12, 15) |
| **The STIckER app seems fitting**, n (%) |  |
| Completely disagree | 0 (0) |
| Disagree | 0 (0) |
| Neutral | 4 (7.8) |
| Agree | 19 (37) |
| Completely agree | 28 (55) |
| **The STIckER app seems applicable**, n (%) |  |
| Completely disagree | 0 (0) |
| Disagree | 0 (0) |
| Neutral | 3 (5.9) |
| Agree | 19 (37) |
| Completely agree | 29 (57) |
| **The STIckER app seems like a good match**, n (%) |  |
| Completely disagree | 0 (0) |
| Disagree | 1 (2) |
| Neutral | 6 (12) |
| Agree | 16 (31) |
| Completely agree | 28 (55) |
| **Feasibility of Intervention Measure (FIM)**, median (IQR) | 20 (16, 20) |
| **The STIckER app seems implementable**, n (%) |  |
| Completely disagree | 0 (0) |
| Disagree | 3 (5.9) |
| Neutral | 5 (9.8) |
| Agree | 12 (24) |
| Completely agree | 31 (61) |
| **The STIckER app seems possible**, n (%) |  |
| Completely disagree | 0 (0) |
| Disagree | 1 (2) |
| Neutral | 4 (7.8) |
| Agree | 16 (31) |
| Completely agree | 30 (59) |
| **The STIckER app seems doable**, n (%) |  |
| Completely disagree | 0 (0) |
| Disagree | 3 (5.9) |
| Neutral | 3 (5.9) |
| Agree | 15 (29) |
| Completely agree | 30 (59) |
| **The STIckER app seems easy to use**, n (%) |  |
| Completely disagree | 0 (0) |
| Disagree | 2 (3.9) |
| Neutral | 4 (7.8) |
| Agree | 12 (24) |
| Completely agree | 33 (65) |

**Number of times intervention providers spoke to patients about STI testing*

**Supplemental Table E8: Table Provider Post Survey: Self-efficacy Questionnaire**

| **Characteristic** | **Overall**  **(N = 41)** | **Control**  **(N = 20)** | **Intervention**  **(N = 21)** |
| --- | --- | --- | --- |
| **Identify the issues the patient wishes to address during the conversation about STIs**, median (IQR) | 8 (6, 9.2) | 8 (8, 10) | 7 (5, 8) |
| **Make a plan for the STI conversation with the patient**, median (IQR) | 8 (7, 9) | 9 (8, 10) | 7 (6, 8) |
| **Urge the patient to expand on his or her problems/worries about STIs**, median (IQR) | 7 (4.8, 9) | 8 (6.5, 10) | 6 (4, 8) |
| **Listen attentively without interrupting or changing focus**, median (IQR) | 8 (6.8, 10) | 9 (8, 10) | 8 (5, 8) |
| **Encourage the patient to express thoughts and feelings**, median (IQR) | 8 (7, 9) | 9 (8, 10) | 7 (5, 8) |
| **Structure the conversation about STIs with the patient**, median (IQR) | 8 (6.8, 9) | 9 (7.5, 10) | 7 (6, 8) |
| **Demonstrate appropriate non-verbal behavior**, median (IQR) | 8 (7, 9) | 9 (8.5, 10) | 7 (6, 8) |
| **Show empathy**, median (IQR) | 8 (7.5, 9.5) | 9 (8, 10) | 8 (6.8, 8) |
| **Clarify what the patient knows in order to communicate the right amount of information about STIs**, median (IQR) | 8 (7, 9) | 9 (7.5, 10) | 8 (6, 8) |
| **Check patient's understanding of the information given about STIs**, median (IQR) | 8 (6, 9) | 8 (8, 10) | 7 (5, 8) |
| **Make a plan based on shared decisions between you and the patient**, median (IQR) | 8 (6.8, 9.2) | 9 (8, 10) | 8 (6, 8) |
| **Close the conversation by assuring that the patient's questions have been answered**, median (IQR) | 8 (7, 9) | 9 (8, 10) | 7 (6, 8) |

**This scale ranged from 10 being very certain to 1 being very uncertain.*
